# Plastome of *Dehaasia pugerensis* Koord. & Valeton: a critically endangered Lauraceae species

**DOI:** 10.3389/fpls.2025.1632459

**Published:** 2026-01-09

**Authors:** Aulia Hasan Widjaya, Andi Salamah, Tety Maryenti, Iyan Robiansyah, Weibang Sun, Mahat Magandhi, Irfan Martiansyah, Muhammad Rifqi Hariri, Aditya Nugroho

**Affiliations:** 1 Department of Biology, University of Indonesia, Depok, Indonesia; 2 Research Center for Applied Botany, National Research and Innovation Agency, Bogor, Indonesia; 3 Research Center for Biota Systems, National Research and Innovation Agency, Bogor, Indonesia; 4 Kunming Institute of Botany, Chinese Academy of Sciences, Kunming, China; 5 Research Center for Biosystematics and Evolution, National Research and Innovation Agency, Bogor, Indonesia

**Keywords:** chloroplast genome, conservation, East Java, endemic species, Indonesia

## Introduction

*Dehaasia pugerensis* Koord. & Valeton is an endemic Indonesian species restricted to Jember Regency, East Java, particularly in the Gunung Watangan area (Bijmoer et al., 2020). This species belongs to the Lauraceae family and has been classified as Critically Endangered (CR) on the IUCN Red List based on criteria B1ab (i, ii, iii, iv, v) and C2a (i) (Helmanto et al., 2022). Its distribution is highly limited, occurring at elevations between 61 and 391 meters above sea level, and its population is threatened by habitat degradation and overexploitation, particularly for charcoal production (Helmanto et al., 2022). These anthropogenic pressures have led to a significant population decline, highlighting the urgent need for science-based conservation efforts, including genomic studies as a foundation for sustainable management of rare species.

One of the key approaches in plant genomics is the characterization of the chloroplast genome. The chloroplast genome in land plants typically exhibits a conserved circular structure, consisting of four major regions: the large single-copy (LSC), the small single-copy (SSC), and two inverted repeat (IR) regions. The total chloroplast genome size in land plants ranges from approximately 120 to 200 kb, with IR regions typically spanning 20–26 kb (Xiao-Ming et al., 2017). In recent decades, advances in sequencing technologies have enabled faster and more accurate assembly of chloroplast genomes. These developments have opened new opportunities to explore the structure, variation, and evolution of chloroplast genomes across plant species, contributing to phylogenetic studies and the conservation of rare genetic resources (An et al., 2022).

To date, the complete chloroplast genome of *D. pugerensis* has not been reported. The absence of such genomic information limits molecular-based conservation initiatives for this species. Within the Lauraceae family, chloroplast genome sizes exhibit considerable variation, as recorded in *Alseodaphne semecarpifolia* (153,051 bp), *Eusideroxylon zwageri* (157,535–157,577 bp), and *Neocinnamomum* spp. (150,753–150,956 bp) (Song et al., 2017; Chen et al., 2017; Li et al., 2017; Cao et al., 2023; Zhu et al., 2023). These data underscore the importance of generating plastome sequences from rare species to deepen our understanding of evolutionary patterns and genetic diversity within Lauraceae. Therefore, this study aims to characterize and reconstruct the complete chloroplast genome of *D. pugerensis* as a foundational resource for genomics-based conservation.

## Methods

### Plant materials

Fresh young leaves were collected from a healthy *D. pugerensis* seedling approximately 1 meter tall, originally sourced from its natural habitat in the Perhutani Forest, Puger, East Java (8°24’10.58” S, 113°30’33.74” E; accession number P.3.1.41). The research and sampling activities were conducted under formal permission from the Perhutani Forestry Institute (PeFI), as stated in Letter No. 0392/001.6/PeFI/2024. The seedling has been conserved at the Cibinong Botanic Gardens (CBG) for *ex situ* conservation purposes.

### DNA extraction, library preparation, and next-generation sequencing

Genomic DNA was extracted from plant leaves using the cetyltrimethylammonium bromide (CTAB) method as described by Doyle and Doyle (1987). The initial DNA concentration and purity were assessed using a Nanodrop 2000 spectrophotometer (Thermo Scientific, MA, USA). DNA integrity was visualized by agarose gel electrophoresis, and quantification was performed with the Qubit dsDNA HS Assay Kit (Thermo Scientific, MA, USA). Further evaluation of DNA integrity was conducted using the 4150 TapeStation system (Agilent Technologies, CA, USA). High-quality genomic DNA was then utilized for library preparation. Sequencing was carried out on the Illumina NextSeq 2000 platform (Genetika Science Lab, Tangerang, Indonesia) employing a paired-end 150 bp strategy, targeting a total data output of 10 Gb.

### Chloroplast genome assembly, annotation and analysis

The reads were assessed for quality using FASTQC software version 0.11.8 (Andrews, 2010). Filtering and trimming were performed using Trimmomatic version 0.39 to remove low-quality bases (less than 30), adapters, nucleotide position bias at the 3’ and 5’ ends, and sequence contamination. The parameters applied included TruSeq3-PE.fa:2:30:10, SLIDINGWINDOW:4:28, LEADING:28, TRAILING:28, and MINLEN:20 (Bolger et al., 2014). The trimmed reads results were subsequently assembled utilizing GetOrganelle version 1.7.7.1 (Jin et al., 2020). The annotation of complete chloroplast genome of *D. pugerensis* was conducted utilizing CPGAVAS2 (http://47.96.249.172:16019/analyzer/annotate) (Shi et al., 2019; Lestari et al., 2024), with the cp genome of *D. hainanensis* (accession number: OP374101.1) serving as the reference. The annotation process faced challenges such as resolving ambiguous gene regions and validating gene boundaries, which were addressed via manual verification using Unipro UGENE v. 45.1 (Okonechnikov et al., 2012) and NCBI Genomic Workbench v. 3.8.2 (Kuznetsov and Bollin, 2021). To ensure the cp genome sequence contained no N bases and had 21 amino acids, Unipro UGENEv. 45.1 was employed. Genes without a start codon were manually edited using the edit menu in NCBI Genomic Workbench v. 3.8.2. The circular genome visualization was performed using Organellar Genome DRAW (OGDRAW) accessed through MPI-MP Chlorobox (Greiner et al., 2019).

### Characterization of simple sequence repeats

Simple sequence repeats (SSRs) of *D. pugerensis* chloroplas genome were identified using the MIcroSAtellite (MISA) web tool (Beier et al., 2017). Search parameters were configured to detect perfect mono-, di-, tri-, tetra-, penta-, and hexa-nucleotide motifs, with minimum repeat thresholds of 10, 5, 3, 3, 3, and 3, respectively. Compound SSRs were allowed when two adjacent repeat motifs were separated by no more than 100 bp.

### Codon usage analysis

Codon usage patterns and Relative Synonymous Codon Usage (RSCU) values were analyzed using MEGA X software (Kumar et al., 2018). Visualization of codon frequency distributions was subsequently performed using the “ggpubr” package in R version 4.2.3.

## Results

**Figure 1:**
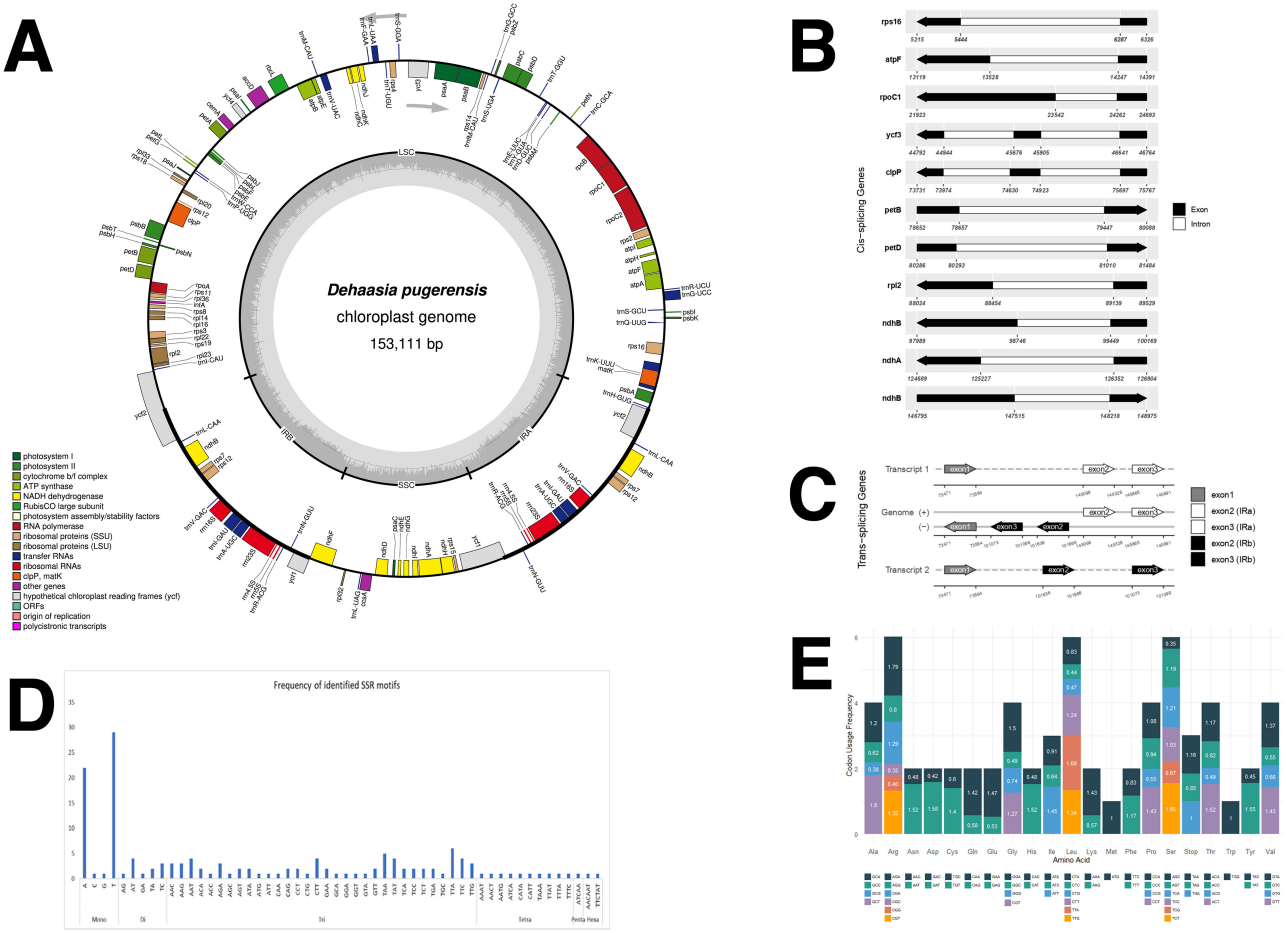
Chloroplast genome features of *Dehaasia pugerensis*. **(A)** Circular chloroplast genome map generated with OGDRAW. Genes on the inner circle are transcribed counterclockwise, while those on the outer circle are transcribed clockwise. Functional gene groups are color-coded, and GC/AT content is shown in grey and light grey. **(B)** Diagram of cis-splicing genes, showing genes that contain introns within a single, continuous locus. **(C)** Diagram of trans-splicing genes, showing genes whose exons are separated across different genomic regions and joined through post-transcriptional splicing. **(D)** Distribution of SSR motif types identified in the chloroplast genome. **(E)** Codon usage patterns for amino acids based on all protein-coding genes in the chloroplast genome.

The complete chloroplast genome of *D. pugerensis* spans 153,111 bp, exhibiting the typical quadripartite structure of angiosperms (**Figure 1A**), comprising a large single-copy (LSC) region of 93,852 bp, a small single-copy (SSC) region of 18,699 bp, and two inverted repeats (IRs) of 20,280 bp each. The GC content is 39.07%, consistent with other Lauraceae species. Genome annotation identified 128 functional genes, including 85 protein-coding genes, 8 rRNA genes, and 36 tRNA genes (**Table 1**). Seven genes are duplicated in the IRs, and 16 genes contain introns, reflecting a level of structural and regulatory complexity typical of Lauraceae plastomes.

**Table 1:** Genes identified in the *Dehaasia pugerensis* chloroplast genome.

Functional category	Group of genes	Names of genes
Self-replication	rRNA	*rrn4.5S*^d^, *rrn5S*^d^, *rrn16S*^d^, *rrn23S*^d^
	tRNA	*trnA*-UGC^d,*^, *trnC*-GCA, *trnD*-GUC, *trnE*-UUC, *trnF*-GAA, *trnG*-GCC, *trnG*-UCC^*^, *trnH*-GUG, *trnI*-CAU, *trnI*-GAU^d,*^, *trnK*-UUU^*^, *trnL*-CAA^d^, *trnL*-UAA^*^, *trnL*-UAG, *trnM*-CAU, *trnfM*-CAU, *trnN*-GUU^d^, *trnP*-UGG, *trnQ*-UUG, *trnR*-ACG^d^, *trnR*-UCU, *trnS*-GCU, *trnS*-GGA, *trnS*-UGA, *trnT*-GGU, *trnT*-UGU, *trnV*-GAC^d^, *trnV*-UAC^*^, *trnW*-CCA, *trnY*-GUA
	Large subunit of ribosom (LSU)	rpl2^*^, *rpl14*, *rpl16*, *rpl20*, *rpl22*, *rpl23*, *rpl32*, *rpl33*, *rpl36*
	Small subunit of ribosome (SSU)	*rps2*, *rps3*, *rps4*, *rps7*^d^, *rps8*, *rps11*, *rps12*^d,e,**^, *rps14*, *rps15*, rps16^*^, *rps18*, *rps19*
	DNA dependent RNA polymerase	*rpoA*, *rpoB*, rpoC1^*^, *rpoC2*
	Subunits of ATP synthase	*atpA*, *atpB*, *atpE*, atpF^*^, *atpH*, *atpI*
	Subunits of NADH-dehydrogenase	ndhA^*^, *ndhB*^d,*^, *ndhC*, *ndhD*, *ndhE*, *ndhF*, *ndhG*, *ndhH*, *ndhI*, *ndhJ*, *ndhK*
Photosynthesis	Subunits of photosystem I	*psaA*, *psaB*, *psaC*, *psaI*, *psaJ*
	Subunits of photosystem II	*psbA*, *psbB*, *psbC*, *psbD*, *psbE*, *psbF*, *psbH*, *psbI*, *psbJ*, *psbK*, *psbL*, *psbM*, *psbN*, *psbT*, *psbZ*, *ycf3*^e,**^
	Subunits of cytochrome b/f complex	*petA*, petB^*^, petD^*^, *petG*, *petL*, *petN*
	Subunit of rubisco	*rbcL*
	Subunit of Acetyl-CoA-carboxylase	*accD*
	C-type cytochrome synthesis gene	*ccsA*
Other function	Protease	*clpP* ^e,**^
	Maturase	*matK*
	Envelop membrane protein	*cemA*
	Translational initiation factor	*infA*
Unknown function	Conserved open reading frames	*ycf1*^d^, *ycf2*^d^, *ycf*4

d = gene duplication, e = three exon, * = intron, ** = double intron.

The conserved structure of the plastome provides a robust molecular framework for phylogenetic resolution within Lauraceae, where morphological convergence often obscures species boundaries (Liu et al., 2017; Tian et al., 2021). Duplicated genes and intron-rich regions offer potential molecular markers for evaluating genetic diversity, population structure, and gene flow (Han et al., 2022). This is critical for *D. pugerensis*, a narrowly endemic and critically endangered species, as chloroplast genomic data inform historical biogeography, demographic shifts, and conservation prioritization (Crawford and Stuessy, 2016).

The chloroplast genome of *D. pugerensis* harbors 11 genes with cis-splicing introns, where exons and introns reside on the same transcript (**Figure 1B**). These include *rps16*, *atpF*, *rpoC1*, *ycf3*, *clpP*, *petB*, *petD*, *rpl2*, *ndhA*, and *ndhB* (the latter two located in IRs and thus duplicated). Gene structures, including exons (black) and introns (white), are annotated with genomic coordinates in the corresponding figure. Most genes, such as *atpF* and *rpoC1*, contain one intron, while *ycf3* and *clpP* have two, indicating complex splicing regulation. Genes located in the IR regions, such as *rpl2* and *ndhB*, appear twice in the genome.

The presence of introns in genes like *clpP* and *ycf3* is conserved among angiosperms and may reflect regulatory or evolutionary functions (Rogalski et al., 2015). The cis-splicing profile in *D. pugerensis* mirrors that of other Lauraceae species, indicating a conserved regulatory mechanism across the family (Song et al., 2017).

The chloroplast genome of *D. pugerensis* contains a single trans-splicing gene, *rps12* (**Figure 1C**). Unlike cis-splicing, trans-splicing joins exons located in separate genomic regions. In *D. pugerensis*, *rps12* is split into three exons: exon 1 resides in the LSC region, while exons 2 and 3 are duplicated in the IRs. These exons are spliced post-transcriptionally to form a functional mRNA. This complex splicing pattern is highly conserved in land plants and is essential for proper chloroplast gene expression. *rps12* encodes a component of the small ribosomal subunit, critical for translation of chloroplast-encoded proteins. Accurate trans-splicing is thus fundamental to chloroplast function and plant development, underscoring the evolutionary significance of maintaining structural and functional integrity in organelle genomes (Oldenburg and Bendich, 2015). The stability of this arrangement also makes *rps12* a reliable phylogenetic marker, offering taxonomic utility in resolving species relationships within Lauraceae (Jacobs et al., 2010; Horiuchi and Aigaki, 2006).

The chloroplast genome of *D. pugerensis* contains 149 simple sequence repeats (SSRs), comprising 53 mononucleotide, 11 dinucleotide, 72 trinucleotide, 10 tetranucleotide, one pentanucleotide, and two hexanucleotide repeats. Trinucleotide repeats—particularly TTA and TAA—are most abundant, followed by A/T-rich mononucleotide repeats (**Figure 1D**). In contrast, the plastome of *D. hainanensis* (NC_068504) is dominated by mononucleotide SSRs with relatively few trinucleotide repeats (n=10), reflecting interspecific variation in SSR profiles (Gao et al., 2018). Such patterns may indicate lineage-specific mutation rates or demographic processes such as historical bottlenecks or prolonged population isolation (Dobrogojski et al., 2020). The elevated proportion of trinucleotide SSRs in *D. pugerensis* may therefore signal unique evolutionary pressures acting on its small, fragmented populations, making these markers valuable for future population-genetic and conservation studies.

Synonymous codon usage analysis in the *D. pugerensis* chloroplast genome provides insights into translational dynamics and selective pressures shaping plastome evolution. Relative Synonymous Codon Usage (RSCU) analysis revealed a distinct codon bias among protein-coding genes (**Figure 1E**). AGA (arginine) showed the highest RSCU value (1.79), while CGG (arginine) had the lowest (0.35), indicating a strong preference among synonymous codons. Leucine was the most abundant amino acid, and tryptophan the least (**Figure 1E**).

A marked bias toward codons ending in A or U was observed, consistent with the AT-rich nature of plastid genomes. Most A/U-ending codons had RSCU > 1, enhancing translational efficiency, while codons such as CGA, GGU, and AGC deviated from this trend. AUG (methionine) and UGG (tryptophan) had neutral RSCU values (1.0), reflecting their lack of synonymous alternatives.

Genomic analyses demonstrate that *D. pugerensis* shares strong plastome conservation with *D. hainanensis*, supporting the need for coordinated conservation strategies across their overlapping native range in East Java. Despite this similarity, distinct SSR profiles and codon usage signatures provide powerful genomic markers for species identification and for evaluating adaptive potential. Combined with ongoing conservation research—including population surveys, DNA barcoding using *rbcL*, *matK, trnH–psbA*, and *ITS* (Widjaya et al., 2025), SSR and ISSR-based genetic diversity assessments, and vegetative propagation—the plastome data strengthen our understanding of evolutionary resilience and inform integrated *in situ* and *ex situ* conservation planning.

Recent conservation efforts have resulted in the collection of 23 seeds and 15 seedlings of *D. pugerensis*, now cultivated at the Bogor Botanic Gardens. These *ex situ* collections serve as essential material for research, propagation trials, and public education (Williams et al., 2015; Westwood et al., 2021). They also support *in situ* recovery efforts by supplying seeds or planting stock for population reinforcement (Heywood, 2017; Abeli et al., 2020). Field assessments by Helmanto et al. (2022) confirm the species’ extremely restricted distribution and high vulnerability, underscoring the need to incorporate genomic evidence into conservation management to enhance long-term survival.

## Data Availability

The original contributions presented in the study are publicly available. This data can be found at the National Center for Biotechnology Information (NCBI) using accession number PQ560536.1. We also wish to inform you that the chloroplast genome sequence of *Dehaasia pugerensis* has been published in NCBI under accession number PQ560536.1 GI: 2844831064. (inc ase 18485643).
